# Can Cancer Education Programs Improve Health Literacy Among Deaf and Hard of Hearing Patients: a Systematic Review

**DOI:** 10.1007/s13187-022-02222-3

**Published:** 2022-09-19

**Authors:** Jan Münstermann, Jutta Hübner, Jens Büntzel

**Affiliations:** 1 Hämatologie Und Internistische Onkologie, Klinik Für Innere Medizin II, Universitätsklinikum Jena, Am Klinikum 1, 07747 Jena, Germany; 2 Klinik Für HNO-Erkrankungen, Kopf-Hals-Chirurgie, Südharz-Klinikum Nordhausen, Nordhausen, Germany

**Keywords:** Cancer, Health literacy, Education, Deaf, Hard of hearing, Communication

## Abstract

Patients affected from hearing loss face many problems when visiting oncologists. We conducted a systematic review to survey if cancer education programs can promote health literacy among deaf and hard of hearing (DHH) patients. The authors searched two databases for RCTs, and cohort studies with interventions promoting cancer health literacy for adult DHH patients. Risk of bias was assessed with SIGN Methodology Checklist for RCTs, and cohort studies. Significance of mean changes over time, and mean differences between comparison groups were used to present outcomes of each study. Surveyed interventions addressed three domains: cancer knowledge, coping skills, and cancer screening. Key information was gathered and synthesized providing a juxtaposition of the content and presenting important effects in detail. Nine RCTs and seven cohorts with 1865 participants were included. In total, 13 studies showed that cancer health literacy interventions improved mean scores significantly from pre- to post-test measures. There are hints that captioning and written texts may be sufficient for milder forms of hearing loss. Three studies showed that resiliency skill training promotes various domains of well-being. Three studies indicated that educational interventions encourage cancer screening practices. Educational programs are an effective way to promote cancer health literacy among DHH patients to facilitate communication with oncologists. As extent of hearing loss was not assessed, the authors cannot say the degree to which results are applicable to all degrees of hearing loss. To obtain hard data, further studies with more diverse populations, various cancer entities, different methods, and exact hearing loss assessments are required.

## Introduction

### Rationale

While congenital hearing loss has mostly genetic reasons, various factors cause acquired hearing loss. The WHO refers to hearing loss as hearing threshold of less than 25 dB. It is categorized into four grades: slight 26–40 dB, moderate 41–60 dB, severe 61–80 dB, and profound > 81 dB hearing loss [1]. Based on the above criteria, the prevalence of hearing loss among adults in Germany is 16.2%. Regarding demographic changes, it is expected to rise [2].

From a functional point of view, the DHH (deaf and hard of hearing) population is heterogeneous with unique communication needs. Deaf individuals are primarily visual processors of information, preferring visual languages and sign language interpreters. Despite their reduced auditory input, hard of hearing individuals are primarily visual-auditory processors and rely on audition and speech. It is expectable that the first group could benefit from a sign language interpreter, while the second could benefit from captions integrated into videos.

DHH patients face many problems when interacting with doctors [3–7]. These obstacles cause low satisfaction with healthcare [8] and have negative impact on patient-provider communication [4, 9]. Although there is much advice on communication with DHH patients [10, 11], it can be difficult to implement these recommendations.

DHH patients struggle for health information access [12] to make health decisions [13]. Cancer-related health literacy interventions for deaf people could facilitate the accessibility of information resources. The findings base on scant studies for specific cancer types and are limited to target populations [14]. Low health literacy hampers effective communication additionally [15, 16]. Promotion of health literacy with tailored programs and meeting could facilitate communication [16, 17]. Regarding direct interactions, we found one systematic review on communication problems of hospitalized DHH patients with doctors. It showed that voice amplifiers are capable of facilitating communication [9]. We identified low health literacy rates among the DHH population as a barrier to effective communication on cancer [15, 16].

### Objectives

Can cancer education programs promote health literacy among DHH patients?

## Materials and Methods

All decisions were unanimously made by the three authors. In case of disagreement, consensus was found by discussion.

### Eligibility Criteria

We used a PICO framework to define eligibility criteria (*Supplementary Material 1*). We included German and English RCTs and cohort studies with interventions improving cancer health literacy among DHH patients, assessed by audiometry or self-report, age ≥ 18 years. Other DHH or hearing patients were accepted as comparisons. We surveyed effects on cancer health literacy, satisfaction with healthcare, and satisfaction with QoL (Quality of life).

### Search Strategy

On 19 September 2021, two medical databases — MEDLINE and EMBASE — were systematically searched. Our search strategy consisted of terms for cancer on the one hand and hearing impairment, sign language, and education programs for the DHH population on the other hand. *Supplementary Material 2* provides our search strings with all restrictions.

### Selection Process

Four steps were taken to select studies for the present systematic review. After detecting doublets, all retrieved titles were screened for relevance. In a third step, remaining records were screened for eligibility. If we could not retrieve full texts, a Google search was launched.

### Data Items

We collected data for change in cancer health literacy. No restrictions were imposed upon assessment methods. One post-interventional assessment was sufficient for inclusion. Data on samples and study designs were gathered. Reported outcomes were summed up in key points.

### Study Risk of Bias Assessment

Risk of bias was assessed with SIGN Methodology Checklists (Version 2.0). The Oxford Centre for Evidence-Based Medicine: Catalogue of Bias [18] was employed to justify a downgrading in the domains of internal validity.

### Effect Measure

We used mean changes in assessment methods to present outcomes. In case of missing data, we summarized tendencies. A *p*-value of ≤ 0.05 was declared the threshold for statistical significance.

### Synthesis Methods

Surveyed interventions address a variety of health literacy programs. The concept of health literacy comprises understanding and using healthcare information [13]. Our structure is derived from these aspects: (1) cancer knowledge, (2) coping skills, and (3) cancer screening. To provide an overview, we created evidence tables comprising five items: intervention, control, assessment method, follow-up, and results. Syntheses of our results are structured in a summary text.

### Certainty Assessment

We assessed certainty of evidence using Oxford Centre for Evidence-Based Medicine: Levels of Evidence and rated studies from levels 1 to 5 [19].

## Results

### Study Selection

Sixteen studies were included in the present review. Our search strategy revealed 961 records in both databases. Checking for duplicates removed 6 hits. After scanning the titles and abstracts for relevance to this research, further 931 publications were excluded. The remaining 24 publications were checked for the prior defined PICO criteria, which lead to the rejection of eight further studies. Reasons for exclusion are shown in Table 1. The process is illustrated under *Supplementary Material 3.*

**Table 1:** Excluded studies

Reference	Reason for exclusion
Berman et al., 2013 [20]	Another study type: cross-sectional study
Brooker et al., 2009 [21]	Another study type: qualitative study
Naseribooriabadi et al., 2018 [14]	Another study type: systematic review
Kushalnagar, Engelman et al., 2018 [22]	Another study type: cross-sectional study
Kushalnagar et al., 2020 [23]	Another study type: cross-sectional study
Peris-Celda et al., 2020 [24]	Another study type: cross-sectional study
Wang et al., 2010 [25]	Another intervention: influence of internal health locus of control (IHLC) on cervical cancer knowledge
Wollin and Elder, 2003 [26]	Another study type: qualitative study

Individual study characteristics are listed in Table 2. Three studies [27, 31, 32] had the same 45 participants. Four studies [31, 32, 37, 42] included all kinds of hearing loss. Four studies had related interventions. Yao et al. [41] used the Choe et al. [28] data of 127 deaf patients to compare them to hearing women. Double patients were not considered in the total count. Sacks et al. [38] surveyed the efficacy of a health literacy program for hearing and deaf patients. Folkins et al. [30] surveyed the same program in comparison to a different prevention program. Participation in the prior study was an exclusion criterion. Each included study reports its own relevant data. Every study assessed hearing loss via self-report.

**Table 2:** Characteristics of included studies

Reference	Participants (intervention [cross-over]/control)	♀/♂	Drop-out (intervention [cross-over]/control)	Mean age (SD)	Assessment of hearing loss	Country
Carter et al., 2021 [27]	45 (24/21)	30/15	16 (1/4) post-intervention (1/10) follow-up	41,33 (14,17)	Self-reported deafness	USA
Choe et al., 2009 [28]	130 (72[56]/58)	130/0	12	41,23 (16,2)	Self-reported deafness	USA
Cumberland et al., 2018 [29]	209 (90/92)	209/0	15 (6/8) follow-up	N/A	Self-reported deafness	USA
Folkins et al., 2005 [30]	102	0/102	7 follow-up	44,35 (17,39)	Self-reported hearing loss	USA
Funes et al., 2019 [31]	45 (24/21)	30/15	16 (1/4) post-intervention (1/10) follow-up	41,33 (14,17)	Self-reported hearing loss	USA
Greenberg et al., 2019 [32]	45 (24/21)	30/15	16 (1/4) pst-intervention (1/10) follow-up	41,33 (14,17)	Self-reported deafness	USA
Harry et al., 2012 [33]	136 (75[61]/61)	68/68	0[6] (0[6] /0) follow-up	37,56 (12,73)	Self-reported deafness	USA
Hickey et al., 2013 [34]	122	122/0	7 follow-up	45,32 (14,19)	Self-reported deafness	USA
Jensen et al., 2013 [35]	107; 52 hearing	107/0	0	55,89 (9,28)	Self-reported deafness	USA
Kaskowitz et al., 2006 [36]	121	0/121	7 follow-up	40,38 (13,91)	Self-reported deafness	USA
Palmer et al., 2017 [37]	150	95/55	Total: 6 (2/0) pre-intervention (3/1) post-intervention	44,5 (14,0)	Self-reported hearing loss	USA
Sacks et al., 2013 [38]	175; 90 hearing	0/175	0	24,18 (4,48)	Self-reported deafness	USA
Sadler et al., 2001 [39]	123	123/0	0	39,3 (14,8)	Self-reported deafness	USA
Shabaik et al., 2010 [40]	144 (86[50]/58)	N/A	13[18] (5[18]/8) follow-up	N/A	Self-reported deafness	USA
Yao et al., 2012 [41]	233; 106 hearing	233/0	0	38,97 (17,51)	Self-reported deafness	USA
Zazove et al., 2012 [42]	195 (97/98)	117/78	156 90 first follow-up 66 s follow-up	55.1 (16.6)	Self-reported hearing loss	USA
Total	1865	1107/614	273	43 (13,8)	-	-

### Risk of Bias in Studies

Five RCTs had a high quality and four an acceptable quality in general minimization of bias. All studies had randomized assignments, adequate concealment methods, comparable treatments, and control groups at the beginning. The only group difference was treatment under investigation. Every study used standard, valid, and reliable outcome measurements. Cumberland et al. [29] did not provide clearly focused questions. They emerge indirectly from the purpose of the surveyed intervention. All RCTs struggled with an adequate blinding. The authors were involved in the development, carrying out, and evaluation. All are affected by a volunteer bias. The participants were invited via flyers to join studies. Five studies [28, 29, 33, 37, 40] used snowball sampling techniques. Every RCT identified education levels as a confounder. Hearing loss was assessed only via self-report. Three controls [28, 33, 40] were primarily inferior. Questionnaires referred to the content of the intervention while the controls were exposed to general cancer information. Cumberland et al. [29] did not provide summarizing statistical analysis. Three studies [27, 31, 32] had a drop-out of 36%. It concentrated on the control group. Zazove et al. [42] had a very high drop-out of 80%. Three studies [27, 31, 32] had small samples (< 100 participants).

All included cohorts had a high quality in general minimization of bias. All included studies had clearly focused questions, defined outcomes, comparable study groups, and reliable methods of assessment. All studies have low risk of attrition bias with a drop-out of less than 20%. Included cohorts are affected by a volunteer bias as patients were chosen through flyers in deaf clubs and snowballing techniques. Hearing loss was assessed via self-report. Three studies [30, 34, 36] assessed prognostic factors multiple times. Three studies [35, 38, 41] compared deaf to hearing cohorts. Education levels were considered as a confounder. All cohorts provided confidence intervals and had an association between exposure and outcome.

*Supplementary Material 4* provides a table with an Oxford Centre for Evidence-Based Medicine: Levels of Evidence [19] rating and a SIGN Methodology Checklists assessment of each included RCT and cohort.

### Results of Individual Studies

The most common endpoint was the gain of cancer knowledge after being exposed to a cancer health literacy intervention. Other outcomes were change in cancer screening behavior and benefits of coping skills. Some adverse events could also be observed. Acquired data are given in Table 3.

**Table 3:** Results of individual studies

	Intervention	Control	Assessment methods	Follow-up	Results
Carter et al., 2021 [27]	d3RP-NF2 (Relaxation, Response, and Resiliency Program for deaf neurofibromatosis type II patients) with information about health topics, and resiliency skill training delivered through videoconferencing	dHEP-NF2 (Health Enhancement Program for neurofibromatosis type II patients) with information about health topics delivered through videoconferencing	PHQ-9 (Patient Health Questionnaire-9) for depression symptom severity PSS-10 (Perceived Stress Scale 10) for perceived stress during the previous month	Baseline Post-intervention (within 1 week after) 6 months	Intervention: Significant reductions in depressive symptoms (*p* ≤ 0.05) and perceived stress (*p* ≤ 0.001). Depressive symptoms remained below MCID (minimal clinically important difference) of 5 points (MCID = 5) Improvements were sustained at follow-up (*p* > 0.1). Depressive symptoms exceeded the MCID of 5 points (MCID = 5.84) Control: No significant improvement in any score at post-test or follow-up
Choe et al., 2009 [28]	Educational video “Cervical Cancer: Catch It Early, and Save Your Life” in ASL with open captioning	National Cancer Institute’s PowerPoint presentation “The Basics” in ASL about cancer in general	8 questions on cancer in general, and 5 on cervical cancer	Baseline Post-intervention 2 months	Intervention: Significant improvement at post-test, and follow-up (*p* ≤ 0.05) Control: No significant improvement Adverse events: Deterioration for one question at post-test (most frequent type of women’s cancer; switch from breast cancer to cervical cancer)
Cumberland et al., 2018 [29]	Educational video about breast cancer in ASL with captioning	PowerPoint presentation, documents, and group discussions about general health topics	5 questions on cancer in general, and 4 on breast cancer	Baseline Post-intervention 12 months	Only relative changes for significantly changed questions (*p* ≤ 0.05) Intervention improved in one more question at post-test, and two more questions at follow-up than control arm Cancer screening: Significant increase in mammography screening, and clinical breast examination at follow-up (*p* ≤ 0.0001) Adverse events: Significant deteriorations for two questions at post-test (correct answers: negations; *p* = 0.016; *p* = 0.007)
Folkins et al., 2005 [30]	Educational video “Prostate, and Testicular Cancer: Know your Options” in ASL with open captioning	–-	11 questions on cancer in general, 7 on testicular, and 7 on prostate cancer	Baseline Post-intervention 2 months	Cohort: Significant improvement at post-test, and follow-up (*p* ≤ 0.05)
Funes et al., 2019 [31]	d3RP-NF2	dHEP-NF2	WHOQOL-BREF (World Health Organization Quality of Life Abbreviated Instrument) for physical, psychological, social, and environmental quality of life (QoL)	Baseline Post-intervention (within 1 week after) 6 months	Intervention: Significant improvement in physical QoL (*p* ≤ 0.001), psychological QoL (*p* ≤ 0.001), social (*p* = 0.001), and environmental QoL (*p* < 0.001) at post-test No significant changes from post-test to follow-up Control: No significant improvement in any domain at post-test Non-significant deterioration for social QoL (*p* = 0.02), no significant changes in other domains at follow-up
Greenberg et al., 2019 [32]	d3RP-NF2	dHEP-NF2	MOCS-A (Measure of Current Status-A) for coping abilities MOS (Medical Outcomes Study Social Support Survey) for social support CAMS-R (Cognitive, and Affective Mindfulness Scale-Revised) for mindfulness GQ-6 (Gratitude Questionnaire) for gratitude LOT-R (Life Orientation Test- Revised) for optimism	Baseline Post-intervention (within 1 week after) 6 months	Intervention: Significant improvement in mindfulness (*p* = 0.008), coping (*p* < 0.001), social support (*p* < 0.001), gratitude (*p* = 0.002), and a non-significant trend for optimism (*p* = 0.079) at post-test, maintained at follow-up (*p* = 0.87) Control: No significant improvement in any score at post-test or follow-up
Harry et al., 2012 [33]	Educational video “Be Smart, Beat Skin Cancer” in ASL with open captioning	Video “Cancer Patients, and Family Support” in ASL	40 questions related to the content of intervention video	Baseline Post-intervention	Intervention: Significant improvement at post-test (*p* ≤ 0.001) Control: No significant improvement
Hickey et al., 2013 [34]	Educational video about breast cancer in ASL with open captioning	–-	10 questions on breast cancer	Baseline Post-intervention 2 months	Cohort: Significant improvement at post-test, and follow-up (*p* ≤ 0.01) Correlation between self-assessed, and actual breast cancer knowledge (*r* = 0.271; *p* = 0.003) Associations for breast cancer knowledge with screening (*p* = 0.005), and mammogram (*p* = 0.07) practices as well as education (*p* < 0.001)
Jensen et al., 2013 [35]	Educational video “Finding, and Surviving Ovarian Cancer” in ASL, and English captioning	–-	29 questions on general, and ovarian cancer	Baseline Post-intervention	Cohort: Significant improvement at post-test (*p* ≤ 0.001) Comparison with hearing patients: Hearing have significantly higher baseline knowledge (*p* < 0.001), and mean increase at post-test (*p* < 0.001) Deaf have significantly higher cancer knowledge at post-test compared to hearing pre-test (*p* = 0.021)
Kaskowitz et al., 2006 [36]	Educational PowerPoint presentation about prostate cancer in ASL	–-	21 questions related to the presentation about prostate cancer	Baseline Post-intervention 2 months	Cohort: Significant improvement at post-test, and follow-up (*p* ≤ 0.05) Trend upwards from post-test to follow-up Cancer screening: Non-significant increase in prostate specific antigen, and rectal exam screenings at follow-up (age 50 +) Adverse events: Deterioration for one question at post-test (“Cancer is a disease that usually passes from parents to children.”, correct answer: negation)
Palmer et al., 2017 [37]	Video about cancer genetics information in ASL with English closed captioning	English text about cancer genetics information, same content	17 questions on cancer genetics, and 8 on genetic counseling	Baseline (within 2 weeks before) Post-intervention (within 2 weeks after)	Intervention/control: Significant improvement at post-test (*p* < 0.001) High education significant improvement (*p* < 0.001) Control: Low education no significant improvement (*p* = 0.79) Intervention: Low education significant improvement (*p* < 0.001) Cancer screening: Significantly more intervention participants feel “very confident” about developing a family tree to identify inherited cancer risk factors (*p* = 0.005)
Sacks et al., 2013 [38]	Educational video “Prostate, and Testicular Cancer: Know your Options” in ASL, and English captioning	–-	4 questions on general cancer knowledge, and 21 on testicular cancer knowledge	Baseline Post-intervention	Cohort: Significant improvement at post-test (*p* < 0.001) Comparison with hearing patients: Hearing have significantly higher baseline knowledge (*p* < 0.001), and mean increase at post-test (*p* < 0.001) Deaf have significantly higher cancer knowledge at post-test compared to hearing pre-test (*p* < 0.001)
Sadler et al., 2001 [39]	Breast cancer education session in ASL with open captioning	–-	7 questions on breast cancer	Baseline Post-intervention	Cohort: Significant improvement for 5 answers at post-test (*p* ≤ 0.05)
Shabaik et al., 2010 [40]	Educational video “Colorectal Cancer: Take Action!” in ASL with open captioning	National Cancer Institute’s PowerPoint presentation “The Basics” in ASL about cancer in general	7 questions on cancer in general, and 13 on colorectal cancer	Baseline Post-intervention 2 months	Intervention: Significant improvement at post-test (*p* ≤ 0.05) Cross-over group had significantly greater improvement at follow-up than original group in CRC knowledge (*p* ≤ 0.05) Control: No significant improvement
Yao et al., 2012 [41]	Educational video “Cervical Cancer: Catch It Early, and Save Your Life” in ASL with English captioning	–-	7 questions on cancer in general, and 5 on cervical cancer	Baseline Post-intervention	Cohort: Significant improvement at post-test (*p* < 0.001) Comparison with hearing patients: Hearing have significantly higher baseline knowledge (*p* < 0.001) Deaf have significantly higher mean increase at post-test (*p* < 0.001)
Zazove et al., 2012 [42]	Educational video about cancer prevention in ASL with captioning	Video with same content in spoken English with captions	12 questions related to the content of the video	Baseline Post-intervention 1 month 6 months	Intervention/control: Significant improvement at post-test (*p* < 0.001), and first follow-up (*p* ≤ 0.01), and second follow-up (*p* ≤ 0.05)

### Results of Syntheses

#### Cancer Knowledge

Six RCTs, and seven cohort studies surveyed the efficacy of cancer health literacy interventions adapted to the needs of DHH patients. Included interventions were adapted through an ASL interpreter and captions. Kaskowitz et al. [36] did not provide any captions. Control interventions were adapted through an ASL interpreter [28, 33, 40], captions [29, 37], and texts [42]. Surveyed programs focused on generic as well as gender-specific cancers. Three studies dealt with breast, two with cervical and two with prostate/testicular cancer. Remaining studies comprised ovarian, prostate, colorectal, and skin cancer as well as cancer genetics and prevention. A total of 1422 DHH patients (167 in cross-overs) were surveyed. 248 hearing patients served as comparisons. All studies assessed hearing loss via self-report and were carried out in the USA. All cohorts and four RCTs had a high quality, two RCTs an acceptable quality.

In case of 12 interventions, the participant’s mean scores improved significantly (*p* ≤ 0.05) from pre- to post-test and to later follow-ups. Cumberland et al. [29] could show that its probands somehow improved but do not provide mean changes. Zazove et al. [42] had long follow-up periods of 1 and 6 months, and Cumberland et al. [29] had an even longer follow-up period of 12 months. Both are affected by high drop-out rates.

Two control groups improved significantly. The Palmer et al. [37] control was exposed to the same content as the intervention but in a written form, not a video in ASL with captioning. Taking education levels into consideration revealed that only the high education group reached comparable results. The low education group improved under intervention conditions only. The Zazove et al. [42] control was exposed to the same video as the intervention without an ASL interpreter, but with captions. They showed that both groups improved equally significantly from baseline to post-test and could retain their knowledge at both follow-ups. Three control arms [28, 33, 40] did not show any significant improvement. In these cases, questionnaires were designed for the intervention. Three cohort studies included hearing individuals [35, 38, 41]. In all cases, the hearing cohort had a significantly higher baseline knowledge. Yao et al. [41] could show higher knowledge increase for the DHH cohort. In both other studies [35, 38], the hearing cohort had higher increase. Still, the DHH post-test scores exceeded hearing pre-test scores.

In three studies [28, 29, 36], deteriorations for some knowledge questions could be observed post-interventional. These effects seem to be caused by confusion and do not affect the overall efficacy of the interventions.

#### Cancer Screening

Three studies with 480 participants surveyed post-interventional cancer screening behavior. Two of these studies had a high [36, 37], and one an acceptable quality [29]. All studies assessed hearing loss via self-reported and were carried out in the USA. Cumberland et al. [29] had the longest follow-up of 12 months and reported a significant post-interventional increase in screening behavior for both groups after 12 months [29]. Kaskowitz et al. [36] found hints at more prostate specific antigen and digital rectal exam screenings for deaf men older than 50 years after 2 months [36]. Palmer et al. [37] detected a significant increase in confidence about developing a family tree to identify inherited cancer risk factors. Actual behavior was not assessed [37].

#### Coping Skills

Three studies [27, 31, 32] report by an example of the same 45 NF-2 patients that adding resiliency skill training to health literacy educational programs reduces depressive symptoms (*p* ≤ 0.01) as well as perceived stress (*p* ≤ 0.001) and improves quality of life (*p* ≤ 0.001) as well as some aspects of resiliency (*p* ≤ 0.01) significantly after intervention. The improvements maintain significant after 6 months. The results are affected by a small sample bias, and a high drop-out, and therefore have an acceptable quality.

### Certainty of Evidence

According to its research designs, nine included RCTs were rated level 2 and seven cohort studies level 3 on the Oxford Centre for Evidence-Based Medicine: Levels of Evidence scale. Cumberland et al. [29] was downrated to level 2 — due to imprecision of its question and missing statistical analysis.

## Discussion

### Interpretation

Inadequate health literacy is one common characteristic of DHH patients. Tailored health literacy programs are one way to meet their individual health needs [15, 16]. Existing data focus on deaf patients and programs including an ASL interpreter. As the DHH population has heterogenous communication needs, strengths, and preferences, it is necessary to develop further formats that are adapted to the needs of hard of hearing people who depend on auditory input. As this target group consists of mainly older individuals with higher incidences of malignant diseases, existing programs could be modified towards their needs.

Our results match closely with previous studies [14]. Educational programs adapted to the needs of the deaf are an effective way to promote cancer health literacy [16]. The limited number of included cancer entities, interventional studies, and small population within the American Deaf community indicate insufficient evidence.

Although most interventions used ASL interpreters to convey information, appropriate English captioning could be sufficient [42]. One study [37] considered education levels in the analysis of their results. The distribution of education levels between both groups was equal but there are no data on correlation between education levels and degree of hearing loss. The control read a text instead of watching a video and comprised more hard of hearing individuals than the intervention: 12% and 4%. A reasonable explanation for these findings could be that the high education group tended to include persons with lesser degrees of hearing loss and therefore relied on texts. This implies that persons more skilled in using written language would benefit primarily from texts or captions, while predominantly visual processors of information would benefit primarily from ASL interpreters. Responding to the heterogeneous communication preferences of DHH persons (text respectively captioning and ASL interpreting) is essential for full accessibility. Adding clear speech to existing programs could be an easy step to meet further individual needs.

However, hearing individuals had higher increases in cancer knowledge in deaf-tailored programs [35, 38, 41]. An explanation may be that deaf audiences find it hard to adopt health information with unfamiliar words and complex grammar [43].

We also identified publications with a new approach. Interventions addressing coping mechanisms for neurofibromatosis type II associated hearing loss provide hints for their efficacy. They are affected by attrition bias as well as very small sample sizes and target at a specific population [27, 31, 32]. Although affected patients seem to profit from this different approach, it is rather unlikely that it influences communication between oncologists and their patients.

Aside from health literacy, we also paid attention to cancer screening rates, a meaningful parameter for the effectiveness of educational interventions. They depict persistence, actual use, and benefit of cancer health education. Three studies could show an increase in screening rates [29, 36, 37]. More data are desirable.

### Limitations of Evidence

All interventions were carried out in the USA and comprise only seven cancer-related topics. Although not all findings obtained in this context might be applicable to different countries, general principles of health education remain the same and can be adapted in other countries and on different topics. Nevertheless, studies including more participants and addressing further topics are desirable. Included interventions concentrated primary on deaf patients. No data were given on the reason and extent of hearing loss as it was measured through self-report. We do not either know, if participants were allowed to use hearing-aids. We cannot say the degree to which results are applicable to milder forms of hearing loss. All included studies are affected by a selection bias due to their sampling techniques and a volunteer bias. They lead to high baseline scores as participants originate from a medically interested population. Apart from Cumberland et al. [29], included studies present complete statistical data. Only three studies surveyed screening rates and one had a long follow-up of 12 months. As it is a long-term parameter surveys presupposes sufficiently long follow-up periods. Longer follow-ups may have led to higher screening rates.

We could not find any data on direct communication strategies for oncologists. A reason for this may be that measures of physician–patient communication reveal mainly poor internal consistency and content validity [44]. A related review on “Impact of Hearing Loss on Patient–Provider Communication Among Hospitalized Patients” demonstrated adverse effects of hearing loss on patient–provider communication and found out that simple bedside interventions such as voice amplifiers improved communication [9].

### Limitations of the Review Process

We excluded all studies with Levels of Evidence [19] below level 3. This might have led to a smaller selection of eligible studies, but we wanted to ground our implications on high certainty of evidence and to know in which extent these data are available. Previous reviews including low certainty data [9] were able to present more eligible studies but found mainly surveys as well as interviews, and only two interventions. We searched two common databases and restricted our results to English and German. Despite these methodological limitations, we are confident to present sound results.

### Implications

We can state that accessible education programs are an effective way to promote cancer health literacy among deaf patients and can therefore have a beneficial influence on oncologist-patient communication. Still, we cannot say the degree to which the effect is dependent on communication preferences and grade of hearing loss. Present data do not include audiometric assessments. Surveyed interventions mostly used ASL interpreters to convey information. It is conceivable that especially milder forms of hearing loss would profit from texts or adequate captioning as it meets their preferred communication mode. Hence, it must be surveyed if given approaches are equally effective for all kinds of hearing loss, and if existing methods could be modified. Further studies with more diverse populations, various cancer entities, different methods, and exact hearing loss assessments are required.

Plenty of advice for physicians on how to interact with DHH patients contrasts with only rudimentary evaluated measures of physician–patient communication. To verify these recommendations and to explore most beneficial communication strategies for DHH patients, further research is strongly recommended.
